# MYO5A overexpression promotes invasion and correlates with low lymphocyte infiltration in head and neck squamous carcinoma

**DOI:** 10.1186/s12885-023-11759-5

**Published:** 2023-12-21

**Authors:** Juanli Xing, Yanan Gu, Yichen Song, Qi Liu, Qian Chen, Peng Han, Zhen Shen, Huajing Li, Shaoqiang Zhang, Yanxia Bai, Junchi Ma, Fang Sui

**Affiliations:** 1 Department of Otorhinolaryngology-Head and Neck Surgery, the First Affiliated Hospital of Xi’an Jiaotong University, 277 Yan-ta West Road, Xi’an, 710061 Shaanxi China; 2 The First Affiliated Hospital of Xi’an Jiaotong University, Xi’an, 710061 Shaanxi Province People’s Republic of China; 3 Department of ophthalmology and otorhinolaryngology, the first hospital in Weinan, No. 35, Shengli Street, Linwei District, Weinan City, 714000 Shaanxi Province China; 4 School of Information Engineering, Chang’an University, Xi’an, 710061 China

**Keywords:** MYO5A, Head and neck squamous carcinoma (HNSC), Immune infiltration, HPV, Metastasis

## Abstract

Head and neck squamous carcinoma (HNSC) poses a significant public health challenge due to its substantial morbidity. Nevertheless, despite advances in current treatments, the prognosis for HNSC remains unsatisfactory. To address this, single-cell RNA sequencing (RNA-seq) and bulk RNA-seq data combined with in vitro studies were conducted to examine the role of MYO5A (Myosin VA) in HNSC. Our investigation revealed an overexpression of MYO5A in HNSC that promotes HNSC migration in vitro. Remarkably, knockdown of MYO5A suppressed vimentin expression. Furthermore, analyzing the TCGA database evidenced that MYO5A is a risk factor for human papillomavirus positive (HPV+) HNSC (HR = 0.81, *P* < 0.001). In high MYO5A expression HNSC, there was a low count of tumor infiltrating lymphocytes (TIL), including activated CD4+ T cells, CD8+ T cells, and B cells. Of note, CD4+ T cells and B cells were positively associated with improved HPV+ HNSC outcomes. Correlation analysis demonstrated a decreased level of immunostimulators in high MYO5A-expressing HNSC. Collectively, these findings suggest that MYO5A may promote HNSC migration through vimentin and involve itself in the process of immune infiltration in HNSC, advancing the understanding of the mechanisms and treatment of HNSC.

**Supplementary Information:**

The online version contains supplementary material available at 10.1186/s12885-023-11759-5.

## Introduction

HNSC are a group of cancers that arise in the mucosal linings of the upper aerodigestive tract, which have an incidence of approximately 600,000 new cases worldwide per year and ranks the 6th most common cancer worldwide [1–3]. Epidemiological evidence indicates that HNSC predominantly affects males and has a fatal outcome in approximately 51% of cases [4]. The management of HNSC typically involves a combination of modalities, including surgical resection, induction chemotherapy, radiotherapy, or a combination of radiotherapy and concurrent chemotherapy, depending on the stage of the disease [5]. Despite the combination therapy approaches available for HNSC, cancers in the head and neck region often exhibit aggressive behavior and are known to poorly respond to both irradiation and chemotherapy. Therefore, patients with locally advanced HNSC typically have a 5-year survival rate of only 50% with the current standard treatment of concurrent chemoradiotherapy [6]. With the improvement of current treatments, the prognosis of head and neck squamous cell carcinoma remains unfavorable. Therefore, novel targeted drugs or other small-molecule–based strategies to the current treatment regimen is eagerly awaited.

The main risk factors associated with the development of HNSCs include tobacco use, excessive alcohol consumption, and infection with oncogenic viruses, such as human papillomavirus (HPV) [7]. It is well known that Chronic usage of tobacco and alcohol has a synergistic effect in disrupting the oral mucosa structure, causing epithelial lesions [8]. Alcohol causes oral epithelial atrophy by interfering with the lipid’s composition of the epithelial layer, hence leading to damage in the DNA synthesis and repair processes, thus contributes to the development of HNSC [4]. While tobacco and alcohol were historically the risk factors for HNSCs, more recently, the proportion of HPV-positive (HPV+) HNSC is projected to become the most common form of head and neck cancer in many developed countries [9]. Which may contribute to different social norms and sexual activities. In the United States, for example, approximately 70% of all oropharyngeal cancers are attributable to HPV [10]. Studies showed that HNSC represent a heterogeneous disease that consists of two clinically distinct entities distinguished by HPV infection [11], showing significantly different survival outcomes and pathway activity [12].

Early studies revealed that Tumor-infiltrating lymphocytes (TILs) have been shown to affect cancer prognosis, and the presence of TIL is associated with improved survival which is increasingly recognized as an important biomarker in [13, 14]. The presence of an inflammatory infiltrate composed of CD8-positive T lymphocytes correlates with improved outcomes in HNSC and this phenomenon may develop because the infiltrated CD8 lymphocytes in the local inflammatory may be available to combat the tumor [15, 16]. Moreover, a higher number of TILs, in particular CD8+ T cells in carcinomas of the head and neck has often been found in HPV+ tumor and stroma, which collectively tend to have a better prognosis than HPV-negative tumors [16–18]. Besides, their function and location of the TILs in the microenvironment appear important and may differ by tumor site and extent in HNSC [19].

In the past decades, little progress has been made in understanding the biological foundation of HNSC, which has significantly hindered the development of more effective treatments. As a result, there is a critical need for novel therapeutic approaches, predictive marker models, and drug delivery systems that can advance the development of effective treatments for HNSC [4]. Indeed, the identification and analysis of genetic aberrations in HNSC samples provide an opportunity for breakthroughs in the development of effective treatments. Oral squamous cell carcinoma, like other head and neck cancers, exhibits a high degree of intratumor heterogeneity, which significantly complicates individualized treatment and directly affects prognosis. Multi-OMICs approaches, which integrate data from DNA mutations, transcriptome, and proteome, have improved our understanding of the molecular mechanisms underlying HNSC and revealed a high degree of inter- and intratumor heterogeneity. Single-cell sequencing techniques have also uncovered RNA-expression signatures related to cell cycle, cell stress, hypoxia, and epithelial differentiation, among others. These findings hold significant promise for advancing the development of novel, more effective treatments for HNSC [20]. For example, studies showed that oral squamous cell carcinoma can be divided into distinct subtypes and these have a preferential response to different types of therapies, suggesting that these gene-based molecular subtype could have clinical implications. Several genes have been found to be involved in the progression of HNSC, however, the role of MYO5A in HNSC remained unclear. Previous studies found that MYO5A mediates melanosome transport and the transport of vesicles to the plasma membrane [21, 22]. In the current study, we examined the expression pattern and functional role of MYO5A by novel bioinformatic data analysis methods and further analyzed the diagnostic and prognostic significance of MYO5A in HNSC tumorigenesis.

## Methods and materials

### Clinical samples

Ethical approval was obtained from the Institutional Review Board and Human Ethics Committee of the First Affiliated Hospital of Xi’an Jiaotong University for this study. To assess head and neck squamous cell carcinoma and para-carcinoma tissue, 172 paraffin-embedded NHSC and para-carcinoma tissue specimens were randomly obtained from the same hospital, with no prior therapeutic interventions administered to the patients. P16 positive and HPV ISH (In situ hybridization) positive were used for HPV−/+ classification. An informed consent was signed before surgery. And both samples were histologically re-examined by two senior pathologists at the hospital’s Department of Pathology. Further details on the baseline characteristics of the HNSC samples can be found in Supplementary Table 1.

### Cell culture experiments and short interfering RNAs (siRNAs) transfection

FaDu cell line was purchased from meisenCTCC, and cultured in DMEM (Dulbecco’s Modified Eagle’s Medium) with 10% FBS and were passaged at 70–80% confluency. The MYO5A -specific and control short interfering RNAs (siRNAs) were purchased from Ribbio. FaDu were transfected with negative control siRNA and MYO5A-siRNA at a final concentration of 25 nM by using Lipofectamine® RNAiMAX Reagent (Life Technologies) 24 hours after plating, according to the manufacturer’s protocol. The details of the siRNA sequences are available in Supplementary Table 2.

### RNA extraction and quantitative RT-PCR (qRT-PCR)

RNA isolation, cDNA preparation and qRT-PCR were performed as described previously [23]. Reverse transcription and qRT-PCR were performed as described previously [24]. Supplementary Table 3 provide the primer sequences used for this study. Each sample was subjected to triplicate runs.

### Immunohistochemical (IHC) staining

Prior to commencing IHC staining, heat-mediated antigen retrieval was performed using Tris/EDTA buffer (pH 9.0). IHC staining utilized an antibody of MYO5A (sc-365,986, Santa) at a 1/300 dilution, followed by a goat anti-mouse IgG H&L(HRP) at a 1/500 dilution. The IHC staining procedure was performed by servicebio Biological Co in accordance with previous descriptions [25] and the expression were quantitated by integral optical density (IOD) using Image-pro plus 6.0 (Media Cybernetics).

### Western blot

Knockdown was confirmed by western blotting at 48 h post-transfection. MYO5A (sc-365,986, Santa) were used for western blot assay at 1/750 dilution, followed by goat anti-mouse IgG H&L(HRP) at 1/100000 dilution. Protein extraction and western blot experiments were performed as previously described [26]. The blots were cut prior to hybridisation with indicated antibodies (MYO5A and TUBULIN) during blotting.

### Bioinformatics analysis

Analyses based on the public database were done with R Statistical Software (4.2.1) and the ggplot2 (3.3.6). Data acquisition: Data were download from the TCGA database (https://portal.gdc.cancer.gov). The RNAseq data of TCGA - HNSC (head and neck squamous cell carcinoma were organize, and the TPM format data and the clinical data were further extracted. Data processing: log2 (value+ 1) was used for analyze. Some Platform was also used for analysis and visualization, and details of each Platform are shown below. CancerSEA (A cancer single-cell state atlas, http://biocc.hrbmu.edu.cn/CancerSEA/home.jsp) [27], cBioPortal (cBioPortal for Cancer Genomics, http://www.cbioportal.org/) [28], TSIDB(The Tumor and Immune System Interactions Database, http://cis.hku.hk/TISIDB/) [29]public online databases were used for extraction of transcriptional information of MYO5As in HNSC and offer distinct functional status of cancer cells at the single cell level. Spearman correlations TISCH2 (http://cis.hku.hk/TISIDB/), UALCAN (http://ualcan.path.uab.edu) [30], TIMER (The tumor immune estimation resource, https://cistrome.shinyapps.io/timer/) [31] public online databases were used for tumor-immune interactions through high-throughput data analysis. KEGG enrichment analysis was performed for internal analysis using the R package clusterProfiler [32]. Significance was determined by the t test and Spearman correlation method and the thresholds were set as *p* < 0.05.

### Statistical analysis

The experiment was repeated at least 3 times. Differences between groups were analyzed using the unpaired Student’s t test or one-way analysis of variance (ANOVA) with SPSS statistical package (16.0, Chicago, IL) or GraphPad Prism 5.0 software. The data were presented as mean ± standard deviation (SD). *P* < 0.05 was considered statistically significant.

## Results

### The expression, methylation and gene mutation of MYO5A in HNSC

**Fig. 1:**
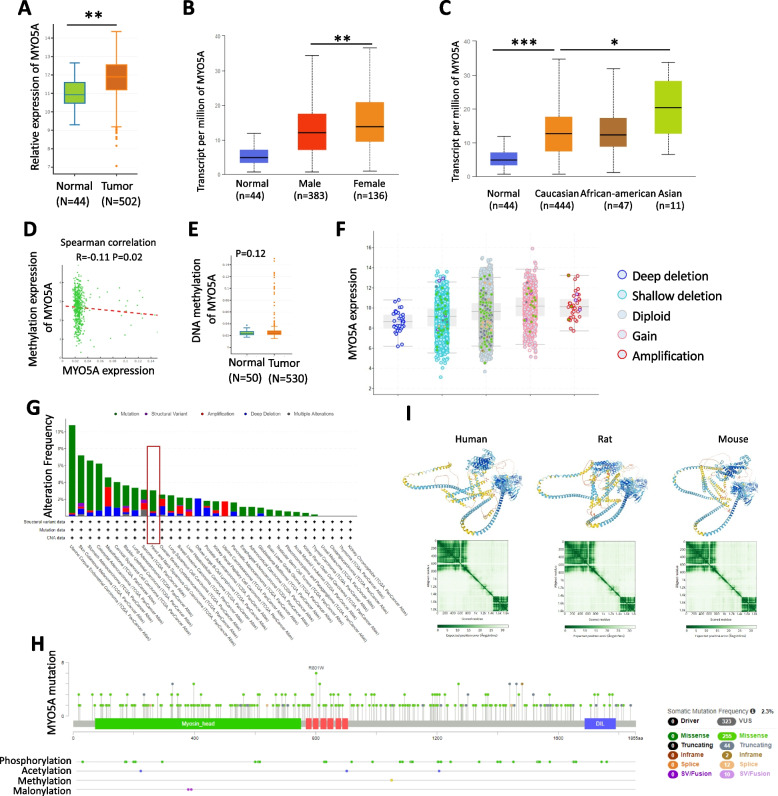
The expression, methylation and gene mutation of MYO5A in HNSC. **A** The transcriptional level of MYO5A in normal tissues and HNSCC (head and neck squamous cell carcinoma) based on TCGA database (TCGA RNAseq data). **B **The differential expression analysis of MYO5A between male and female HNSCC patients was conducted using UALCAN based on TCGA database. **C** The differential expression analysis of MYO5A in HNSCC patients with different race (Caucasian, African-american and Asian) was conducted using UALCAN based on TCGA database. **D** Spearman correlation analysis was made between DNA methylation and gene expression of MYO5A in HNSCC patients according to TCGA database. **E** DNA methylation level of MYO5A in normal tissues and HNSCC based on TCGA database. **F** The copy number alterations (Deep deletion, Shallow deletion, Diploid, Gain and Amplification) and expression levels of MYO5A in HNSCC patients using cBioportal. **G**-**H** The overall mutational spectrum of MYO5A in pan-cancer were performed by cBioportal based on TCGA database. **I** 3D protein structure prediction and their expected prediction error of MYO5A in human, mouse and rat were performed by AlphaFold Protein Structure Database. The colour at position (x, y) indicates AlphaFold’s expected position error at residue x, when the predicted and true structures are aligned on residue y. AlphaFold produces a per-residue confidence score (pLDDT) between 0 and 100. Some regions below 50 pLDDT may be unstructured in isolation. Data are presented as mean ± SD. *, *P* < 0.05; **, *P* < 0.01, ***, *P* < 0.001

Using TCGA RNAseq data, we confirmed the higher expression of MYO5A in HNSC than in normal tissue (Fig. 1A). Besides, the expression of MYO5A was higher in female HNSC patients compared with male patients (*P* < 0.01, Fig. 1B). And the expression of MYO5A is higher in Asian than other ethnic (e.g. Caucasian, African- american), shown in Fig. 1C. Spearman correlation between gene expression and methylation of MYO5A in HNSC (Fig. 1D). However, there was no statistically significant difference of MYO5A methylation between HNSC sample and normal tissue (Fig. 1E). According to the TCGA pan-cancer Atlas studies, the copy-number alterations, such as gain or amplification of the MYO5A indeed leads to higher expression of this gene when compared with deep or shallow deletion (Fig. 1F). With regard to mutation, the overall mutational spectrum of MYO5A in pan-cancer were performed by cBioportal based on TCGA database (Fig. 1G-H). 3D protein structure prediction and their expected prediction error of MYO5A in human, mouse and rat were performed by AlphaFold Protein Structure Database (Fig. 1I).

### MYO5A promotes migration and invasion of HNSC

**Fig. 2:**
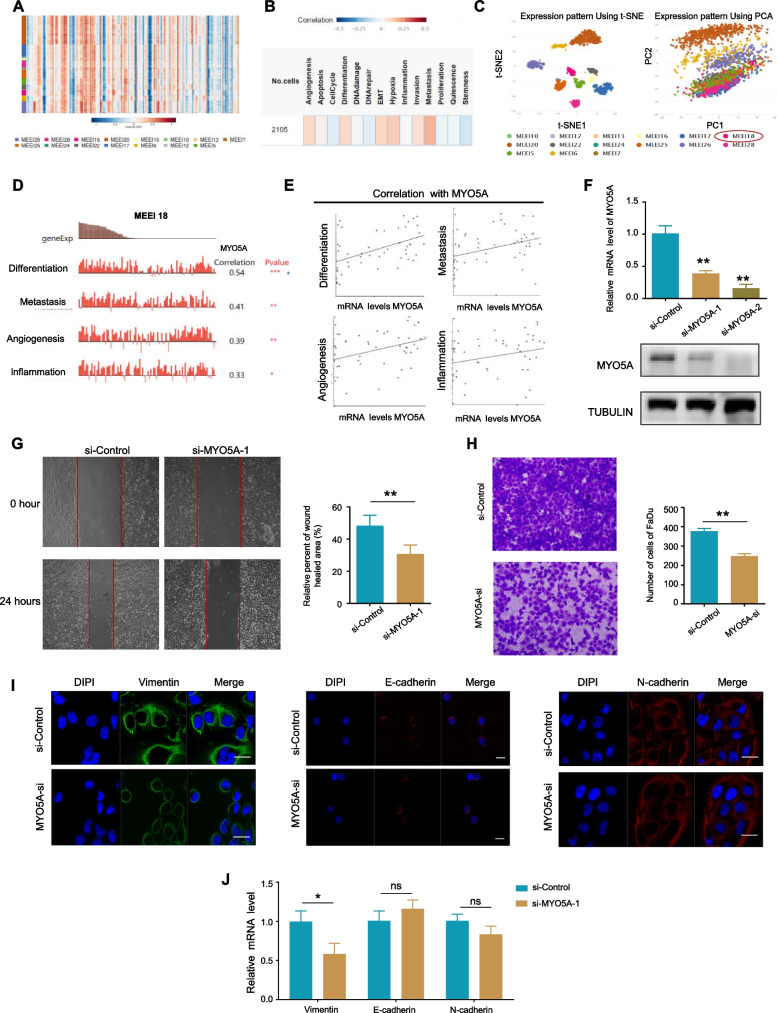
MYO5A promotes migration and invasion of HNSC. **A** A heatmap showing the Copy Number Variation (CNV) profile of MYO5A gene in different clustered cells in HNSC using single-cell RNA-seq data from GSE103322 using CancerSEA. Rows represent hierarchically clustered cells, columns represent PCGs which ordered by genomic location (1-22 and X), and separation represents the gap of chromosomes. **B** Correlations between the MYO5A and functional states in HNSC using single-cell RNA-seq data from GSE103322. **C** The expression pattern of MYO5A by t-SNE (t-distributed stochastic neighbor embedding) and PCA (principal component analysis) analysis in HNSC (GSE103322). **D** Functional states that are significantly related to MYO5A in cluster MEEI 18 in HNSC (GSE103322). **E** The correlation of differentiation, metastasis, angiogenesis and inflammation with MYO5A. **F** Efficiency of siRNA knockdown of MYO5A in FaDu cell line was performed by RT-PCR (at 36 hours post-transfection) and western blot (at 48 hours post-transfection). The blots were cropped from different parts of the same gel prior to hybridisation with different antibodies. Tubulin was used as the reference. **G**-**H** Cell migration and invasion were respectively assessed by wound healing and chamber cell migration assays. At 36 hours post-transfection, FaDu cells were seeded for migration and invasion assays. **I** Expressions of Vimentin, E-cadherin and N-cadherin were examined by Immunofluorescence staining. FaDu cells were seeded for Immunofluorescence staining assays at 48 hours post-transfection. **J** mRNA of Vimentin, E-cadherin and N-cadherin were further examined by RT-PCR assay. FaDu cells were seeded for Immunofluorescence staining assays at 36 hours post-transfection. Data are presented as mean ± SD. *, *P* < 0.05; **, *P* < 0.01, ns, no significant difference

Using single-cell RNA-seq data of HNSC (GSE103322), the Copy Number Variation (CNV) profile of MYO5A gene in different clustered cells in HNSC was showed in a heatmap (Fig. 2A). To obtain further insights into the linkage between the expression and potential function of MYO5A, correlations analysis between the MYO5A and functional states in HNSC was performed, using single-cell RNA-seq data from GSE103322. The result showed that MYO5A may promote the metastasis (highest level of correlation) in HNSC (Fig. 2B). Further analysis using t-SNE (t-distributed stochastic neighbor embedding) and PCA(principal component analysis) in the cluster MEEI18 (with the largest statistically significant differences) showed a correlation between MYO5A expression and differentiation (R = 0.54, *P* < 0.001), metastasis (R = 0.41, *P* < 0.01), angiogenesis(R = 0.39, *P* < 0.01), and inflammation (R = 0.33, *P* < 0.05) shown in Fig. 2C-E. To examine whether MYO5A facilitates metastasis in HNSC, knockdown experiments were conducted in the FaDu HNSC cell line. RT-PCR and western blot assays demonstrated the efficacy of siRNA knockdown of MYO5A at 36- and 48- hours post-transfection, respectively (Fig. 2F). Wound healing and chamber cell migration assays were used to respectively assess cell migration and invasion at 36 hours post-transfection in FaDu cells, showing that knockdown of MYO5A decreased the in vitro migration and invasion activity of FaDu cells (Fig. 2G-H). Immunofluorescence staining for EMT marker genes, including Vimentin, E-cadherin, and N-cadherin, was also performed, revealing that MYO5A knockdown impaired the expression of Vimentin but had no significant effect on E-cadherin or N-cadherin expression in FaDu cells (Fig. 2I). RT-PCR assay was performed to confirm these findings (Fig. 2J).

### MYO5A associated with poor prognosis in HNSC especially in HPV+ HNSC

**Fig. 3:**
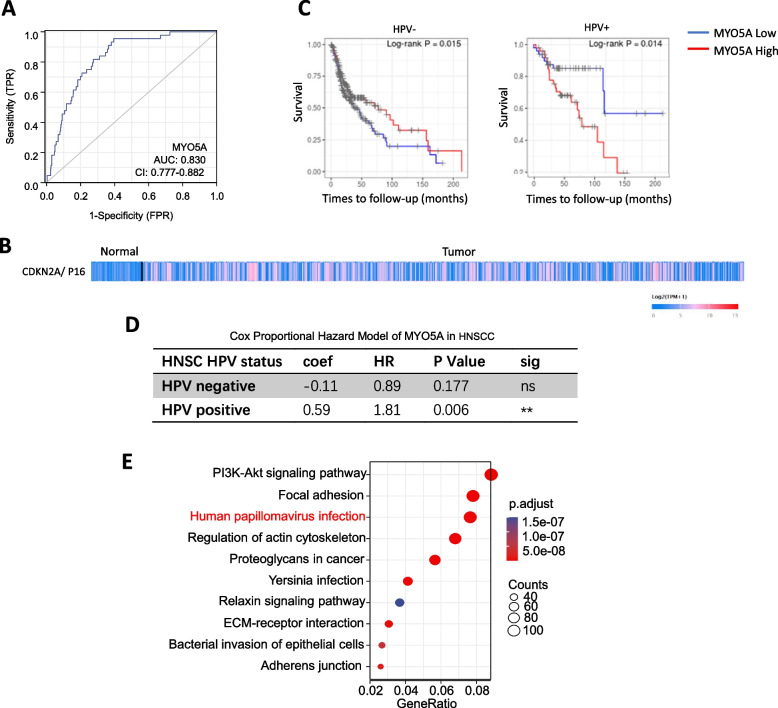
MYO5A associated with poor prognosis in HNSC especially in HPV+ HNSC. **A** The ROC analysis of MYO5A in HNSC based on clinicopathological parameters from TCGA. **B** DKN2A/P16 expression is represented as a heat map, with shades of red representing up-regulation and shades of blue indicating down-regulation based on TCGA database by UALCAN. **C** The Kaplan-Meier survival curve compare samples stratified according to MYO5A expression levels in HPV- and HPV+ HNSCC. **D** Cox Proportional Hazard Model of MYO5A in HNSCC based on TCGA database by Timer platform. **E** KEGG pathway enrichment analysis (www.kegg.jp/kegg/kegg1.html) of MYO5A in HNSC. *P* < 0.01; ns, no significant difference

An ROC analysis based on clinicopathological parameters from TCGA was employed to investigate the diagnostic potential of MYO5A in HNSC. The resulting Area Under the Curve (AUC) = 0:83 suggests that MYO5A can be considered a reliable biomarker for differentiating HNSC cases (Fig. 3A). It has been widely reported that infection with HPV is a risk factor for HNSC, however, HNSC patients with HPV-positive (HPV+) cases typically exhibit better responses to therapy, more favorable prognoses, and extended overall survival compared to HPV-negative cases (HPV-) [33]. CDKN2A/P16 positivity is often used as a marker of HPV infection. CDKN2A/P16 expression In HNSC and normal tissue is represented as a heat map, with shades of red representing up-regulation and shades of blue indicating down-regulation (Fig. 3B). Interestingly, high levels of MYO5A were associated with poorer prognoses in HPV+ HNSC cases, however, there is better survival with high MYO5A levels in the HPV negative group (*p* = 0.015). (Fig. 3C). The Cox Proportional Hazard Model also demonstrated a statistically significant HR = 1.81 (Hazard ratio, *p* = 0.006) for MYO5A in HPV+ HNSC cases, whereas the association was not statistically significant for HPV- cases (HR = 0.89, *p* = 0.177), shown in (Fig. 3D). Furthermore, KEGG pathway analysis revealed human papillomavirus (HPV) infection to be linked to MYO5A in HNSC (Fig. 3E). Besides, survival curve was also built in HNSC with high or low MYO5A expression according to cancer site, including Oral tong (*n* = 133), Larynx (*n* = 116), Floor of mouse (*n* = 64), Oral cavity (*n* = 73) and Tonsil (*n* = 46), shown in Supplementary Fig. 1.

**Fig. 4:**
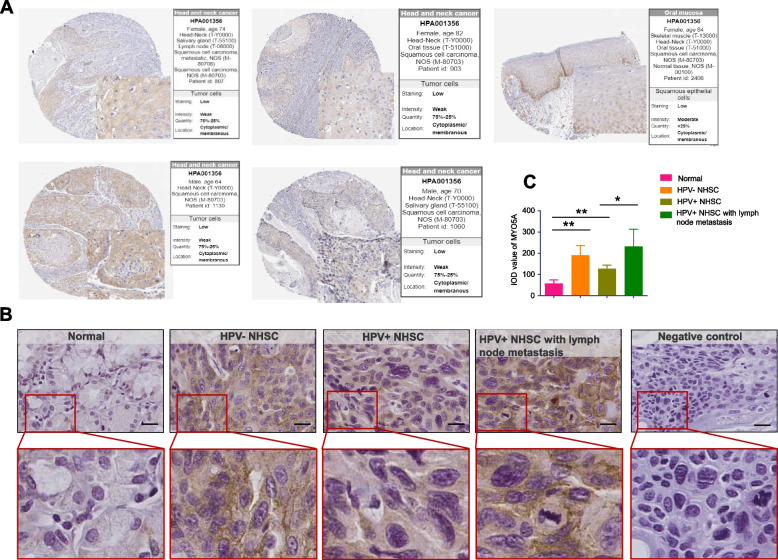
MYO5A expression in HNSC tissues. **A** Atlas database showed MYO5A expression in HNSC tissues (https://www.proteinatlas.org/). **B** The expression of MYO5A in HPV- HNSC (*n* = 22), HPV+ HNSC (*n* = 5), HPV+ HNSC with lymph node metastasis (*n* = 16) and normal tissue (*n* = 21) by immunohistochemistry (IHC) analysis on whole tissue sections. **C** Statistical analysis based on the IOD value of Fig. 4B. *, *P* < 0.05. **, *P* < 0.01. Bar = 25 μm

The Atlas database was utilized to illustrate the expression patterns of MYO5A in HNSC tissues (Fig. 4A). In addition, immunohistochemistry (IHC) analysis was conducted to confirm the expression of MYO5A protein in HPV-HNSC, HPV + HNSC, adjacent normal tissues, and HPV+ HNSC (with metastasis). The IHC results revealed amplification of MYO5A in HNSC relative to normal tissue. IHC results showed increased levels of MYO5A in HNSC compared with normal tissue. Moreover, metastatic HPV + HNSC exhibited heightened MYO5A expression in comparison to HPV + HNSC without metastasis (Fig. 4B-C).

### MYO5A was associated low immune infiltrate in HNSC

**Fig. 5:**
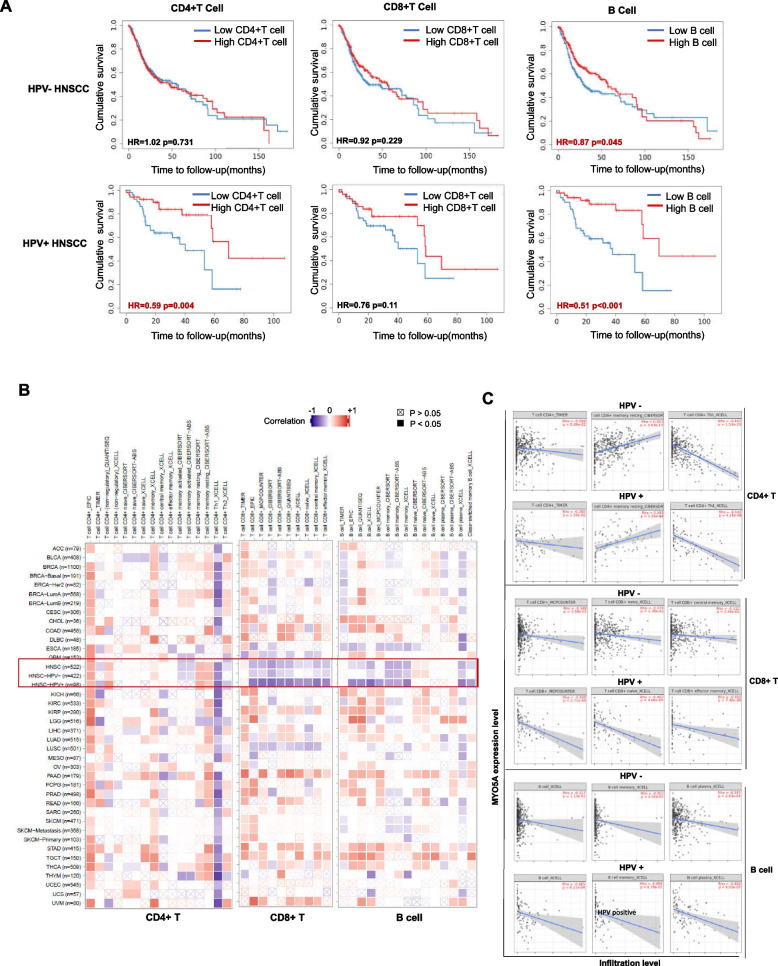
The association of MYO5A with immune infiltration in HNSC. **A** Kaplan–Meier survival curve for the abundance ratios of tumor-infiltrating immune cells (CD4+ T cells, CD8+ T cells and B cells) in HPV+ and HPV- HNSC. TIMER, Tumor Immune Estimation Resource. **B** The associations between tumor immune infiltrating cells (CD4+ T cells, CD8+ T cells and B cells) and MYO5A in HNSC samples were evaluated using the TIMER database

**Fig. 6:**
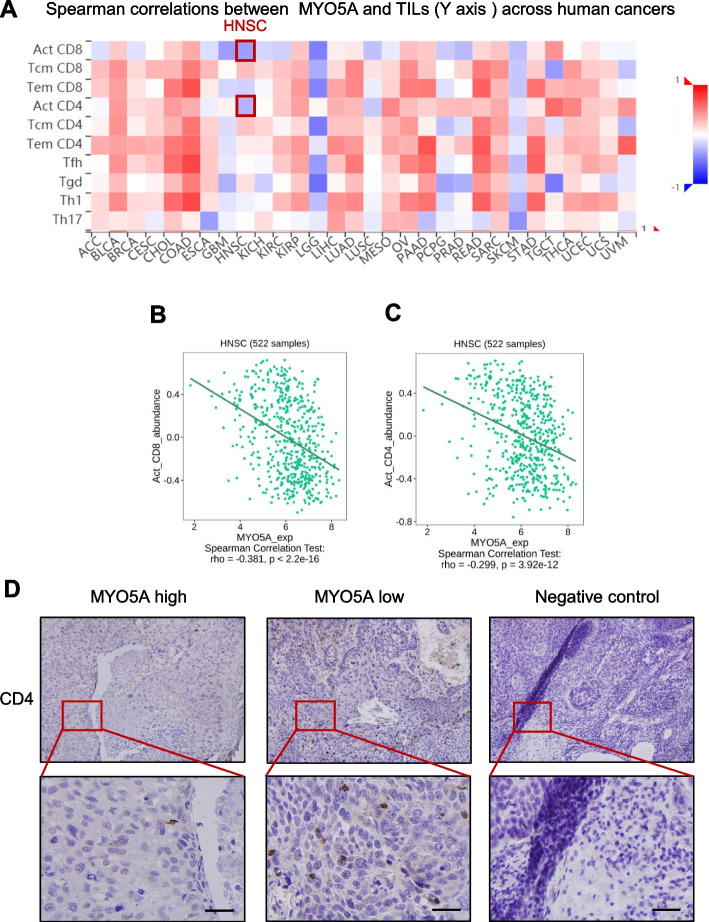
The correlation of MYO5A and TILs. **A-C** The correlation analysis of MYO5A and TILs was also performed using gene set variation analysis (GSVA) based on gene expression profile through TISIDB platform. **D** The expression pattern of CD4 was assessed in HPV+ HNSC cases with high or low MYO5A expression by immunohistochemistry (*N* = 21)

In addition to tumor intrinsic factors, emerging evidence suggests that patterns of infiltrating immune cell types also contribute to tumor progression in HNSC [34]. To preliminarily investigate the impact of infiltrating immune cells on HNSC prognosis, we performed Kaplan-Meier survival analyses using TCGA database data and found that elevated levels of infiltrating CD4+ T cells (HR = 0.59, *P* = 0.004) and B cells (HR = 0.51, *P* < 0.001) correlated with longer survival in HPV+ HNSC cases, but not in HPV- HNSC cases (*P* > 0.05) (Fig. 5A). To further explore the relationship between MYO5A expression and immune infiltration patterns in HNSC, we utilized the TIMER 2.0 online tool to analyze expression data from pan-cancer samples. Our analyses revealed that MYO5A expression was significantly and inversely correlated with the infiltration levels of CD4+ T, CD8+ T cells and B lymphocyte cells in HNSC (Fig. 5B). To augment these results, we employed gene set variation analysis (GSVA) using gene expression data from TISIDB platform to investigate the correlation of MYO5A with tumor infiltrating lymphocytes (TILs) in HNSC. This analysis revealed an inverse correlation between MYO5A expression and activated CD8 T cell (R = -0.38, *P* < 0.001) and activated CD4 T cell (R = -0.29, *P* < 0.001) infiltrates in HNSC (Fig. 6 A-C). Consistently, found samples with high MYO5A showed lower CD4+ T cell infiltration (Fig. 6 D) in HPV+ HNSC cases by immunohistochemistry.

Single-cell sequencing analysis using GSE103322 and GSE139324 datasets revealed that MYO5A expression is present in functionally distinct subpopulations with varying subcellular localizations within single cells, including Immune cells and stromal cells (Supplementary Fig. 2). Considering that some genes are implicated in regulating TIL by inflammation-regulation and cytokine secretion, we performed a correlation analysis to investigate the interaction between MYO5A and immunostimulators in pan-cancer using TISIDB. Additionally, MHCs showed a negative correlation with MYO5A expression based on TCGA database samples (Supplementary Fig. 3). These findings suggest that MYO5A may play a role in modulating immune infiltrates in HNSC.

### Gene enrichment analysis and downstream analysis of MYO5A in HNSC

**Fig. 7:**
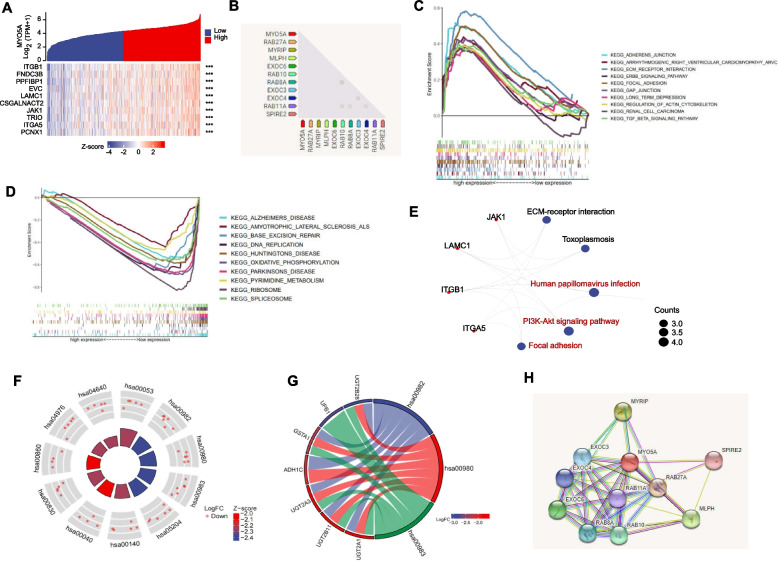
Gene enrichment analysis and downstream analysis of MYO5A in HNSC. **A** Heat maps of mRNA expression levels for high- and low-MYO5A-expressing in A-HNSC samples based on TCGA database. Principal component analysis was performed with the software R (4.2.1) and was visualized with “ggplot”. **B** The top 10 genes with the strongest association with MYO5A was listed. **C-D** Gene Set Enrichment Analysis (GSEA) of MYO5A were performed using the GSEA software (version 3.0). **E** Correlations were tested by Spearman’s correlation coefficient and five top KEGG categories were identified by R Statistical Software. **F** KEGG enrichment analysis of MYO5A were performed by R Statistical Software. **G** The circle graph of the DEGs of MYO5A in HNSC. **H** PPI network of MYO5A-associated genes was performed by STRING. DEG, Differentially expressed genes

Molecular pathways associated with MYO5A expression in HNSC were investigated using differential gene expression analysis, GSEA, and PPI network construction. Heat maps were used to display mRNA expression levels for high- and low-MYO5A-expressing TCGA-HNSC samples (*p* < 0.001; Fig. 7A), with the top 10 genes showing the strongest association with MYO5A identified, including RAB27A, MYRIP, MLPH, EXOC6, RAB10, RABBA, EXCO3, EXCO4, RAB11A, and SPIRE2 (Fig. 7B; *p* < 0.01). GSEA analysis with both up-regulated and down-regulated GSEA hallmark gene sets or pathways resulted in the identification of altered pathways in response to MYO5A expression (Fig. 7C-D). Furthermore, the top five KEGG categories of the five molecules with the highest Spearman correlation coefficients with MYO5A expression were identified (Fig. 7E). KEGG enrichment analysis revealed that DEmRNAs were enriched in the Human papillomavirus infection, PI3K-Akt signaling pathway, and Focal adhesion pathways. The upregulated DEmRNAs were also found to be enriched in the Ascorbate and aldarate metabolism (KEGG ID: hsa00053), Drug metabolism-cytochrome P450 (KEGG ID: hsa00982), and Metabolism of xenobiotics by cytochrome P450 (KEGG ID: hsa00980) pathways (Fig. 7F and Supplementary Table 4). The circle graph illustrated that the differentially expressed genes (DEGs) including UGT2B28, UPB1, GSTA1, ADHIC, UGT2A3, UGT2B11, and UGT2A1 were enriched in the top three KEGG categories (Fig. 7G). Finally, a PPI network of 10 co-expressed MYO5A-associated genes was constructed using STRING (Fig. 7H).

## Discussion

This study observed that the inhibition of MYO5A genes resulted in the significant inhibition of cell migration and invasion of FaDu cells. Furthermore, vimentin expression was observed to be downregulated upon MYO5A knockdown. Known for its ability to regulate cytoskeletal organization in various cancer cell types, including HNSC, vimentin is an important protein in tumor invasion and migration. Changes in vimentin expression potentially reflect the molecular mechanisms that play a role in the migration and invasiveness of FaDu cells. The findings suggest that MYO5A perhaps suppresses the migration and invasion of HNSC cells by indirectly downregulating vimentin in vitro.

This study conducted a Kaplan-Meier survival analysis, which demonstrated a statistically significant shorter survival rate in the MYO5A-high group, as compared to the MYO5A-low group, in HPV-positive HNSC samples. Furthermore, we defined MYO5A as a risky gene (HR =1.81, *P* < 0.006) for HPV-positive HNSC samples, after employing Cox proportional hazard model analysis of MYO5A in HNSC. Previous studies showed that HNSC represent a heterogeneous disease that consists of two clinically distinct entities distinguished by HPV infection [11] and the mutational makeup of HPV+ and HPV- HNSC differs significantly [35]. These findings suggest that MYO5A may be responsible for some of the variance and may trigger a worse prognosis in HPV+ HNSC. Enrichment analysis indicated that MYO5A had a correlation with HPV. We also observed an overexpression of MYO5A in HPV+ HNSC with metastasis, when compared to none-metastatic cases. Taken together, these results indicate that MYO5A likely plays a role in HPV infection and progression of HPV+ HNSC. However, the direct impact of MYO5A genes on HPV infection will require further exploration. p16/CDKN2A is a surrogate marker for HPV infection and is overexpressed when the E7 protein binds to pRb, thereby releasing the E2F transcription factor in HPV-infected cells [36]. In KEYNOTE-012, for the head-and-neck cohorts, when stratified by p16 status, response rates were higher in p16 + patients compared to p16- patients, with demonstrated ORRs of 24% (95% CI, 13–40%) and 16% (95% CI, 10–23) respectively [37, 38]. These results insist that certain HPV-related biomarkers may have a crucial role in predicting prognosis and stratifying patients for adjuvant treatment in HNSC. Considering the aberrant oncogenic potential of HPV (low-risk and high-risk types), the description of HPV genotype variants could also be of interest.

Interestingly, studies revealed a higher number of TILs, in particular CD8+ T cells in HPV+ HNSC tumor, which collectively tend to have a better prognosis than HPV-negative tumors [16–18]. We observed that patients with high MYO5A expression had reduced immune cell infiltration, especially CD8+ T cells and B cells, as well as a concomitant decrease in immunostimulators in HNSC. Research has shown that the microenvironmental characteristics influence the response to immunotherapy. For instance, treatment with checkpoint inhibitory drugs, such as PD-1, may have more effectiveness in tumors with a more pronounced lymphocytic infiltrate [39]. More recently, with the approval of checkpoint inhibitors for the treatment of cancers including oral squamous cell carcinoma(OSCC), genomics studies also dissected the genetic signatures of the immune compartment to delineate immune-active and -exhausted subtypes and guide the development of novel therapies to improve response to immunotherapy [40]. Research is also investigating innovative therapeutic approaches, such as gene therapy, and immunotherapy [4]. New targets is being explored, involved in the way how tumor cells interact with stroma cells and the immune cells [41]. The above results suggested that MYO5A may exert a specific function in immune infiltration of HNSC. and may hamper the efficacy of immunotherapy.

This study proposes differential expression and the promoting effect of MYO5A on the migration and invasion of HNSC, but it has some limitations that need addressing. Firstly, some bioinformatics analyses based on TCGA database have not yet been verified by other independent databases. Nonetheless, we verified some of the results with our molecular biology experiments to strengthen the validity of our findings. Secondly, this study primarily focused on correlation analysis, and biostatistical correlations alone cannot elucidate direct interactions and regulatory mechanisms. As a result, our future experiments aim to explore the interactions of various molecules in HNSC and understand the potential mechanisms.

### Supplementary Information

**Additional file 1: Supplementary Figure 1.** Survival curve with high or low MYO5A expression in different subtype of HNSC. **Supplementary Figure 2.** Single cell sequencing analysis of MYO5A in HNSC. **Supplementary Figure 3.** The correlation analysis of MYO5A and MHCs. **Supplementary Table 1.** A summary of the baseline characteristics of HNSC patients (*N*=172). **Supplementary Table 2.** SiRNA sequences used in this study. **Supplementary Table 3.** The primers used for qRT-PCR assays. **Supplementary Table 4.** The enriched KEGG functions of significantly upregulated DEmRNAs of MYO5A.

**Additional file 2.**

## Data Availability

Data were download from the TCGA database- HNSC (head and neck squamous cell carcinoma (https://portal.gdc.cancer.gov). All data generated or analyzed during this study are included in this published article [and its supplementary information files].
