# Supplementary material for: MYO5A overexpression promotes invasion and correlates with low lymphocyte infiltration in head and neck squamous carcinoma
Source: BMC Cancer. 2023 Dec 21;23:1267. doi: 10.1186/s12885-023-11759-5 (PMC10740236; doi:10.1186/s12885-023-11759-5)
Supplement: Supplementary file 1 — Additional file 1: Supplementary Figure 1. Survival curve with high or low MYO5A expression in different subtype of HNSC. Supplementary Figure 2. Single cell sequencing analysis of MYO5A in HNSC. Supplementary Figure 3. The correlation analysis of MYO5A and MHCs. Supplementary Table 1. A summary of the baseline characteristics of HNSC patients (N=172). Supplementary Table 2. SiRNA sequences used in this study. Supplementary Table 3. The primers used for qRT-PCR assays. Supplementary Table 4. The enriched KEGG functions of significantly upregulated DEmRNAs of MYO5A. [file 12885_2023_11759_MOESM1_ESM.docx]

Supplementary Material

MYO5A overexpression promotes invasion and correlates with low lymphocyte infiltration in head and neck squamous carcinoma

**Juanli Xing^1+^, Yanan Gu^1+^, Yichen Song^2^, Qi liu^3^, Qian Chen^1^, Peng Han^1^, Zhen shen^1^, Huajing Li^1^, Shaoqiang Zhang^1^, Yanxia Bai^1^, Junchi Ma^4^*, Fang Sui^1^***

*Correspondence:

E-mail (Fang Sui): sf_brighteyes@163.com

E-mail (Junchi Ma): majunchi@chd.edu.cn

# Supplementary Figures and Tables

## Supplementary Figures


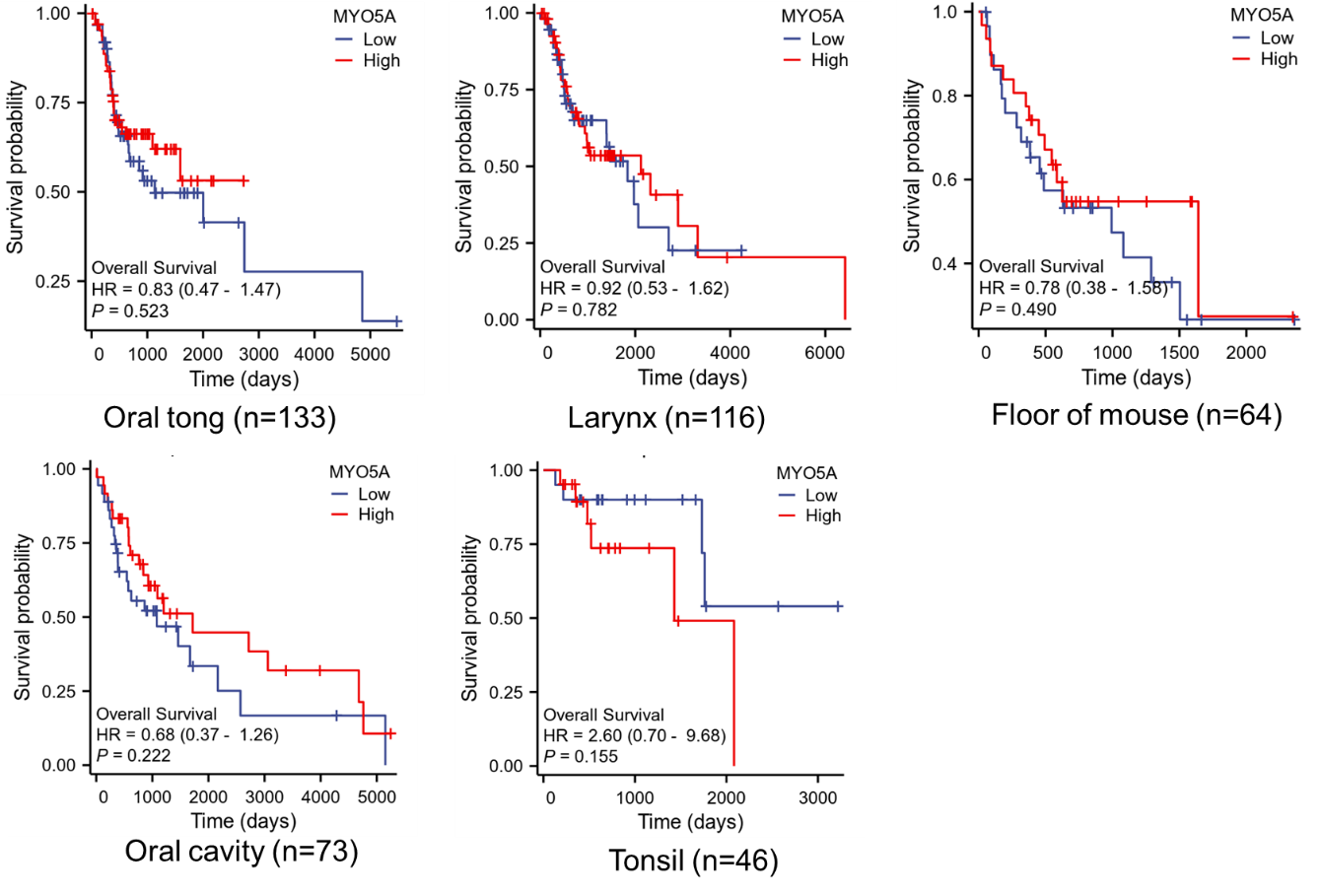


**Supplementary Figure 1.** **Survival curve with high or low MYO5A expression in different subtype of HNSC.** Survival curve was also built in HNSC with high or low MYO5A expression according to cancer site, including Oral tong (n=133), Larynx (n=116), Floor of mouse (n=64), Oral cavity (n=73) and Tonsil (n=46), based on TCGA database.


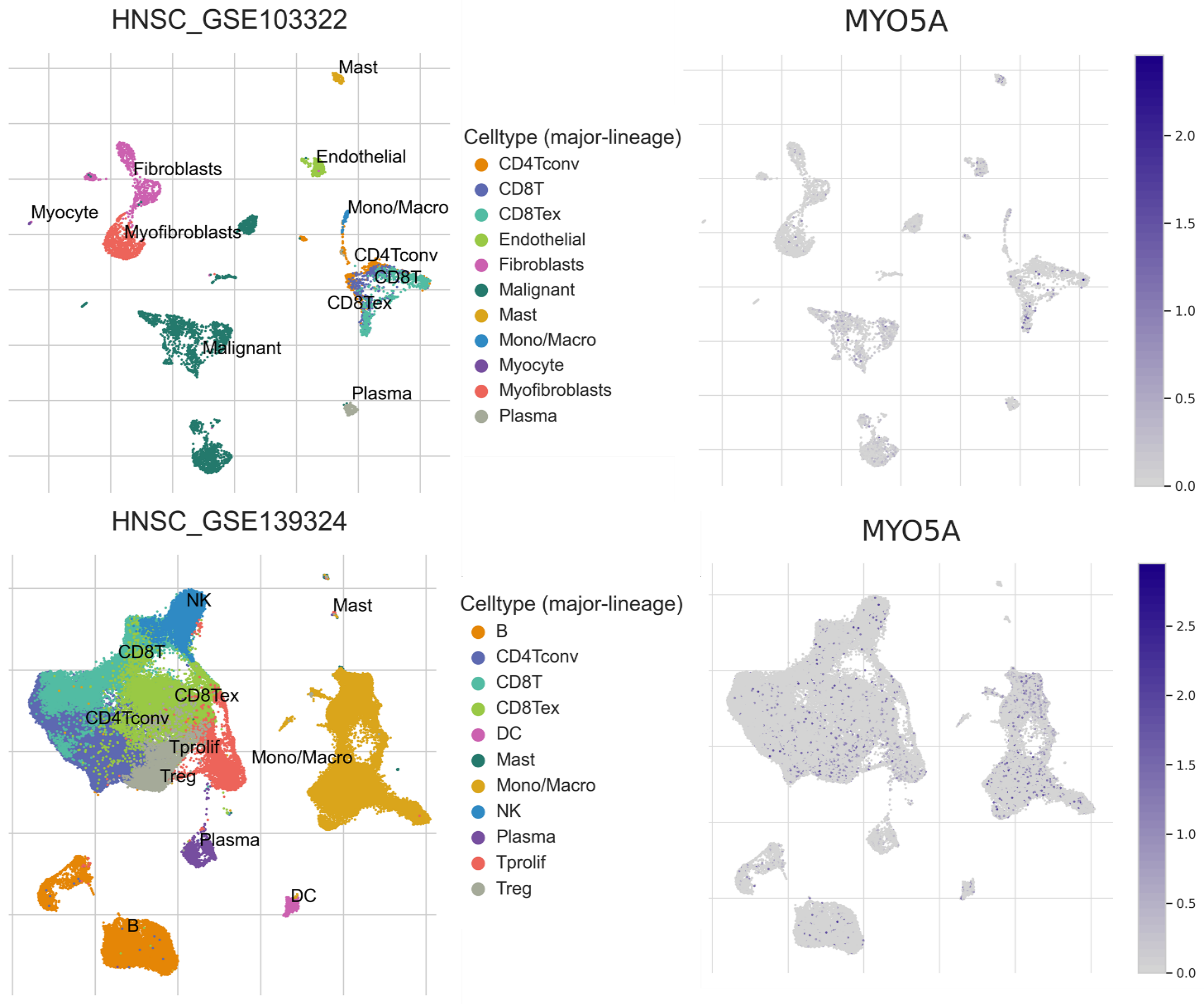


**Supplementary Figure 2.** **Single cell sequencing analysis of MYO5A in HNSC.** Single cell sequencing analysis of MYO5A in HNSC bases on GSE103322 and GSE139324 database (http://tisch.comp-genomics.org/).


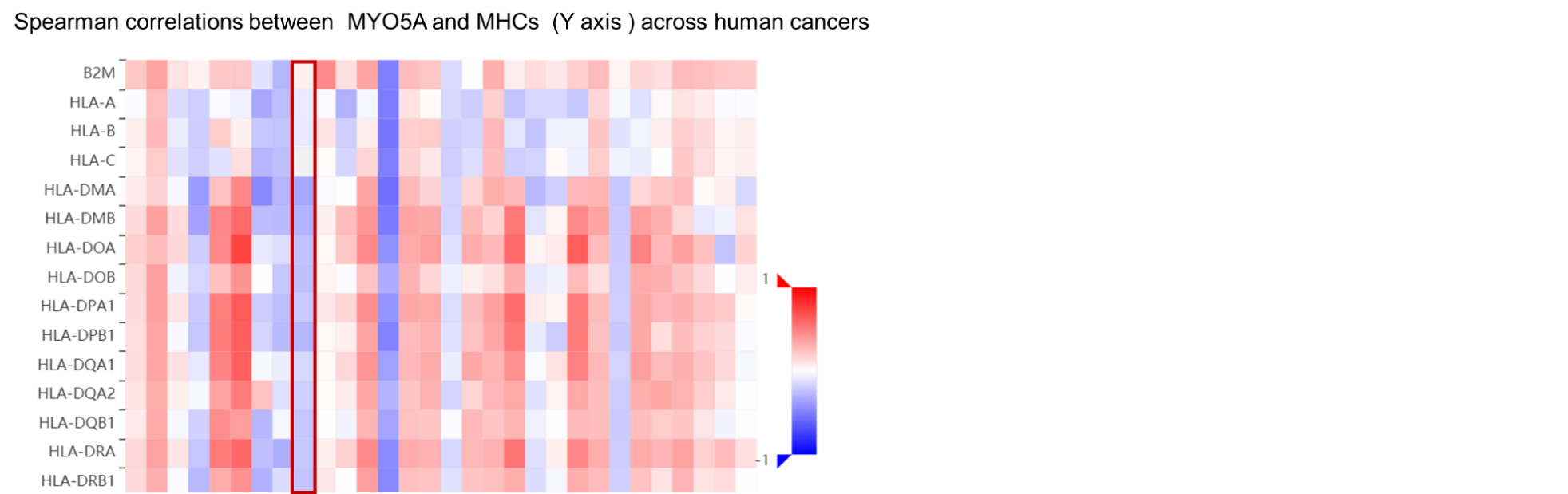


**Supplementary Figure 3.** **The correlation analysis of MYO5A and MHCs.** The correlation analysis of MYO5A and MHCs was also performed using gene set variation analysis (GSVA) based on gene expression profile through TISIDB platform (http://cis.hku.hk/TISIDB/).

## Supplementary Tables

**Supplementary Table 1**. A summary of the baseline characteristics of HNSC patients (N=172).

| Characteristics | HNSC | Percentage (%) |
| --- | --- | --- |
| **Gender** |  |  |
| Male (number) | 121 | 73.3 |
| Female (number) | 51 | 29.7 |
| **Age** |  |  |
| Age (year) | 38-87 | / |
| Mean age (year) | 60.7 | / |
| **T stage** |  |  |
| T1 | 15 | 8.7 |
| T2 | 43 | 25.0 |
| T3 | 50 | 29.1 |
| T4 | 64 | 37.2 |
| **N stage** |  |  |
| N0 | 13 | 7.6 |
| N1 | 28 | 16.3 |
| N2 | 117 | 68.0 |
| N3 | 14 | 8.1 |
| **HPV status** |  |  |
| OPC (-) | 49 | 28.5 |
| OPC (+) | 21 | 12.2 |
| Not OPC | 102 | 59.3 |

OPC, Oropharyngeal carcinoma; HPV, human papillomavirus

**Supplementary Table 2**. SiRNA sequences used in this study.

| si-RNAs | Sequences (5′-3′) |
| --- | --- |
| si-MYO5A-1 | GGATTGTAGATAATGTCAATC |
| si- MYO5A-2 | CGCTACAAGAAGCTCCAT |

**Supplementary Table 3**. The primers used for qRT-PCR assays.

| Genes | Sequences (5′-3′) |
| --- | --- |
| Human-MYO5A(sense) | CGGAAAGACCTGGAGCAAACTC |
| Human- MYO5A (antisense) | TGCTGCACGATGCGGTGATTGA |
| Human-Vimentin (sense) | AGGCAAAGCAGGAGTCCACTGA |
| Human- Vimentin (antisense) | ATCTGGCGTTCCAGGGACTCAT |
| Human- E-Cadherin (sense) | GCCTCCTGAAAAGAGAGTGGAAG |
| Human- E-Cadherin (antisense) | TGGCAGTGTCTCTCCAAATCCG |
| Human- N-Cadherin (sense) | CCTCCAGAGTTTACTGCCATGAC |
| Human- N-Cadherin (antisense) | GTAGGATCTCCGCCACTGATTC |
| Human -18S (sense) | CGCCGCTAGAGGTGAAATTC |
| Human -1*8S* (antisense) | CTTTCGCTCTGGTCCGTCTT |

**Supplementary Table 4.** The enriched KEGG functions of significantly upregulated DEmRNAs of MYO5A.

| Ontology | ID | Description | Gene Ratio | Bg Ratio | P value | p.adjust | qvalue |
| --- | --- | --- | --- | --- | --- | --- | --- |
| KEGG | hsa00053 | Ascorbate and aldarate metabolism | 5/79 | 30/8076 | 9.28e-06 | 0.002 | 0.001 |
| KEGG | hsa00982 | Drug metabolism - cytochrome P450 | 6/79 | 71/8076 | 6.25e-05 | 0.005 | 0.004 |
| KEGG | hsa00980 | Metabolism of xenobiotics by cytochrome P450 | 6/79 | 77/8076 | 9.87e-05 | 0.005 | 0.004 |
| KEGG | hsa00983 | Drug metabolism - other enzymes | 6/79 | 79/8076 | 1.14e-04 | 0.005 | 0.004 |
| KEGG | hsa05204 | Chemical carcinogenesis | 6/79 | 82/8076 | 1.40e-04 | 0.005 | 0.004 |
| KEGG | hsa00140 | Steroid hormone biosynthesis | 5/79 | 61/8076 | 3.06e-04 | 0.008 | 0.007 |
| KEGG | hsa00040 | Pentose and glucuronate interconversions | 4/79 | 34/8076 | 3.15e-04 | 0.008 | 0.007 |
| KEGG | hsa00830 | Retinol metabolism | 5/79 | 68/8076 | 5.08e-04 | 0.011 | 0.010 |
| KEGG | hsa00860 | Porphyrin and chlorophyll metabolism | 4/79 | 42/8076 | 7.16e-04 | 0.014 | 0.012 |
| KEGG | hsa04976 | Bile secretion | 5/79 | 90/8076 | 0.002 | 0.031 | 0.028 |
| KEGG | hsa04640 | Hematopoietic cell lineage | 5/79 | 99/8076 | 0.003 | 0.043 | 0.038 |
