# Supplementary figures and images for: MYO5A overexpression promotes invasion and correlates with low lymphocyte infiltration in head and neck squamous carcinoma
Source: BMC Cancer. 2023 Dec 21;23:1267. doi: 10.1186/s12885-023-11759-5 (PMC10740236; doi:10.1186/s12885-023-11759-5)

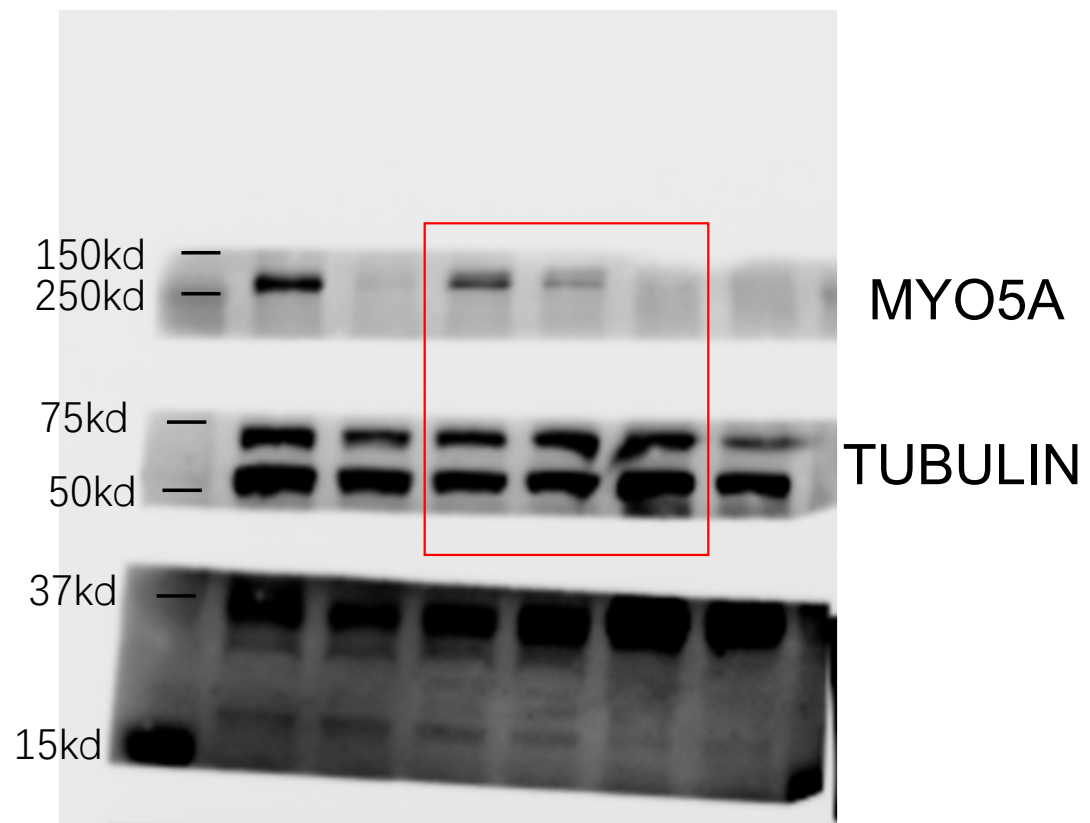

Gels and blots with whole membrane edges

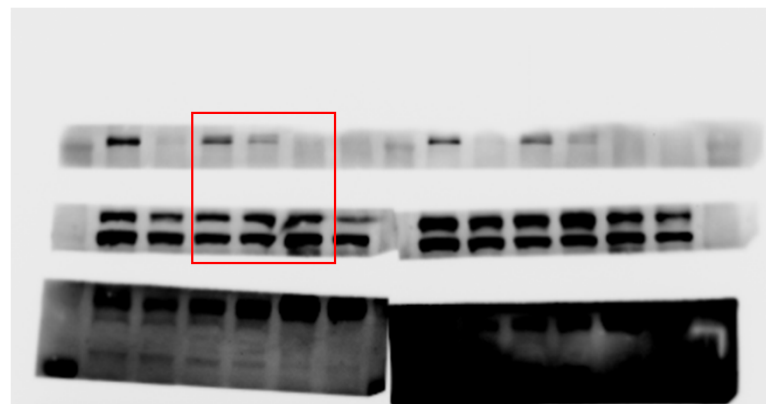

Supplement: Supplementary file 2 — Additional file 2. [file 12885_2023_11759_MOESM2_ESM.pdf]
